# Follow the Money—The Politics of Embryonic Stem Cell Research

**DOI:** 10.1371/journal.pbio.0030234

**Published:** 2005-07-12

**Authors:** Eugene Russo

## Abstract

Gene Russo examines the broader implications of Proposition 71 - a California initiative to fund and promote research into human embryonic stem cells.

German embryonic stem cell scientist Oliver Brustle faces major challenges in his lab on a daily basis that have little to do with science. His country's policy on human embryonic stem cells (hESCs) is among the most restrictive in Europe. Collaborations with other countries are difficult. And he's overwhelmed by the time and paperwork necessary to comply with regulations and to apply for permits.

Brustle, an investigator at the University of Bonn (Bonn, Germany), often ponders the possibility of taking his work elsewhere. “I think about it every day,” he says. “Sometimes [the regulations] get to the point where they're suffocating.” Many of his German colleagues have similar sentiments, he says. In Germany, scientists can only work on hESC lines derived in labs outside the country, and even then, only if the line was derived prior to 2002. Federal law prohibits the derivation of new hESC lines.

Yet Brustle is optimistic that hESC technology, flush with research dollars in many countries and garnering interest worldwide, is destined for greatness. He takes solace in the fact that his long-time scientific pursuit has potential for success on several different fronts. Indeed, though some hESC scientists like Brustle are frustrated, others are thriving.

The most recent front is California, where Proposition 71 [1], passed last November, sent science policy shock waves across the United States and around the world (Box 1). Promising an impressive US$3 billion in funding for hESC research over the next ten years, “Prop 71” could further complicate an already complex landscape of laws and funding sources (assuming supporters can prevail over legislation that has stalled the measure). Other states in the US are struggling to catch up, setting the stage for a patchwork of policies and funding efforts.

**Box 1. Proposition 71**

California's Proposition 71 calls for US$3 billion of stem cell research funding over 10 years. The money will be raised by general fund bonds. The measure includes the establishment of the California Institute for Regenerative Medicine (http://www.cirm.ca.gov/) to regulate and oversee stem cell research, a mission that entails managing ethical research practices and awarding grants through peer review (peer reviewers will be based outside the state of California). The Institute's 29-member Independent Citizens Oversight Committee consists of representatives from California universities, nonprofit institutions, patient advocacy groups, and the biotechnology industry. Grant reviews will be by out-of-state scientists to prevent conflicts of interest. Unlike the policy dictated by President Bush in August 2001, Prop 71 permits the funding of the derivation of new human embryonic stem cell lines, including via SCNT research (therapeutic cloning). Currently, 78 hESC lines around the world are eligible for federal US funding, but only 22 have been developed into distribution-quality cell lines. With interest, Prop 71 is projected to cost the state approximately US$6 billion if paid over a 30-year period. Lawsuits attempting to stop Prop 71 are currently pending, according to a spokesperson for the California Institute for Regenerative Medicine. It's not yet clear, she said, for how long the litigation will stall the sale of bonds and the awarding of research dollars.

Countries from Europe to Asia also have a patchwork of different policies (Figure 1), as economic and scientific interests clash with religious and cultural mores in a battle over research that has yet to help a single patient. And while many non-Californian stem cell researchers are cheering the Prop 71 windfall, they're wary of brain drain to California in a field that already has a shortage of talent. Some supporters also worry that a mosaic of state laws will make collaborations more difficult and future therapies hard to administer. In short, Prop 71 is likely to help shape the future of stem cell research in the US and around the world.

**Figure 1:**
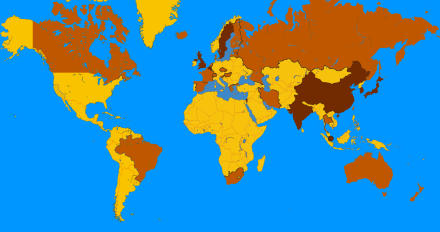
Stem Cell Policy Map Countries colored in brown have a permissive or flexible policy on human embryonic stem cell research. All have banned human reproductive cloning. These countries represent about 3.4 billion people, more than half the world's population. “Permissive” (countries in dark brown) means that various embryonic stem cell derivation techniques are permitted, including SCNT. “Flexible” (countries in light brown) means that stem cells may be derived from human embryos donated by fertility clinics only, excluding SCNT. Countries in yellow have either a restrictive policy or no established policy. (Image: William Hoffman, MBBNet)

## An Intriguing Proposition

Stanford University (Stanford, California, United States of America) stem cell scientist Irv Weissman, who played a key role in the multimillion dollar effort that recruited researchers, advocates, and Hollywood actors to support Prop 71, attributes the effort's inception to a 2002 US National Academy of Sciences report on cloning. It contended that therapeutic cloning, or somatic cell nuclear transfer (SCNT), could be used to generate disease-specific stem cell lines; the research had more future applications than just cell and organ transplantation (Box 2). Scientists might be able to actually revert diseased cells to their primordial form and then monitor them to see how and why abnormalities develop. That notion sparked interest from many people unhappy with President Bush's policy, which dictates that no federal funding may be used to work on hESC lines derived after August 2001. (On May 24 the US House of Representatives passed a bill that would make more cell lines available for federal funding. Bush has vowed to veto the bill.) Also of concern: stem cell lines approved by the National Institutes of Health (NIH) (http://stemcells.nih.gov/research/registry/), which can only be grown on a layer of mouse feeder cells, would not be appropriate for clinical use since animal viruses could theoretically jump to humans. According to some, the president's policy was simply too restrictive.

**Box 2. Sources of hESCs**

Researchers primarily obtain human embryonic stem cells from frozen embryos donated by IVF programs. Cell lines may be grown by isolating hESCs from the inner cell mass of a human blastocyst, a five-day-old embryo. These cells are cultured indefinitely with the help of fibroblast feeder layers. Several groups are now attempting to grow feeder-free cell lines, which would enable investigators to know exactly what molecular factors are contributing to hESC growth [2]. According to Austin Smith, director of the Institute for Stem Cell Research at the University of Edinburgh, if scientists can discover the minimum requirements for self-renewal, they'll be able to better control and direct robust hESC growth, which could be advantageous for both basic and clinical hESC research.

Researchers may also obtain hESCs via SCNT, as was demonstrated last year by a group in South Korea [3]. The technique involves removing the nucleus, and hence the nuclear genome, of an oocyte and replacing it with the nucleus of an adult cell. The activated egg can then form a blastocyst, which contains identical genetic material to that of the donor adult cell, and this is the process often referred to as therapeutic cloning. By using SCNT rather than a donated IVF embryo, researchers would be able to control the genotype of hESCs, which could help circumvent tissue rejection problems in future clinical applications as well as help investigators study the development of diseases via disease cell lines. In May, Hwang et al. became the first to derive patient-specific human embryonic stem cells from SCNT blastocysts [4]. Even when countries allow SCNT, lawfully securing donated oocytes can be a challenge. Last year, Hwang et al. [3] used 200 oocytes before deriving a single cell line. His more recent study [4] was much more efficient. His group's work has yet to be widely repeated. If implanted in a uterus, a blastocyst generated via nuclear transfer could theoretically develop into a human being genetically identical with the nuclear donor.

And then Hollywood got involved. Producers Janet and Jerry Zucker, who have a child with diabetes, asked Weissman to educate them about stem cells, which led to the formation of an advocacy organization called CuresNow (Los Angeles, California, United States of America). State Senator Debra Ortiz took up the cause, and suggested that California support research where the NIH could not. Soon after, the politically savvy Robert Klein, a board member of the Juvenile Diabetes Foundation, began spearheading the Prop 71 effort. (Klein has a child with diabetes as well.) Weissman and other scientists conducted dozens of talks, educating the public about stem cells and cautioning them about the potentially slow pace of hESC research. Governor Arnold Schwarzenegger decided to endorse the plan. And in November of last year, voters voiced their approval.

Though some states already had initiatives under way, Prop 71 spurred and accelerated efforts. Many states are now engaged in a race to attract stem cell research with laws and regulations that defy the Bush administration policy. In some cases, states are pushing for funding packages to offset the federal funding shortfall and insure that their most promising scientists don't head west. And in other cases, states are reacting strongly in the opposite fashion, considering major restrictions on the sort of stem cell research that scientists in their state can conduct.

“As this issue has hit the state level, there has been a noticeable change in the terms of the debate” says Daniel Perry, executive director of Coalition for the Advancement of Medical Research (Washington, District of Columbia, United States of America), which represents patient organizations, universities, scientific societies, and foundations. Before Proposition 71, moral status questions were at the fore, e.g., debates as to when life begins and how society should balance the rights of a potential life with those of a person suffering from a life-threatening disease. “Now the debate is all about economic development,” says Perry. “Are we going to be creating jobs in Delaware or Illinois or Pennsylvania or are we going to be losing them to states next door that are in the stem cell business?” Stem cell research supporters fear their states, many with a history of biotechnology and life sciences investment, will lose talent, dollars, and infrastructure to California or other states if they don't protect their own research investment.

## State by State

More than 30 US states are considering some sort of legislation related to stem cell research, for or against. Particularly active, according to Perry, are Wisconsin, Illinois, New York, Delaware, Texas, Florida, Washington, and Missouri. In Illinois, the state house and senate are considering a bill that imposes a surcharge on elective cosmetic surgery as a way to raise an estimated US$1 billion for stem cell research over the next ten years. In New Jersey, with the support of Governor Richard Cody, the legislature approved US$150 million in state funding to build a Stem Cell Institute. New Jersey citizens will vote on a US$230 million bond this November that would finance stem cell research over the next seven years. Connecticut recently approved $100 million for stem cell research.

The senate and house in Massachusetts recently voted to explicitly allow all aspects of stem cell research, including work on surplus embryos and SCNT. Legislators had enough votes to override Massachusetts governor Mitt Romney's veto. “For those of us worried that everything could get shut down, this is a big relief,” says Leonard Zon, a Howard Hughes Medical Institute (Chevy Chase, Maryland, United States of America) investigator and the director of the stem cell research program at Children's Hospital Boston (Boston, Massachusetts, United States of America). Zon, who works on deriving blood stem cells from embryonic stem cells, spent considerable time helping to support the legislation. Massachusetts has some high-profile stem cell researchers, and many feared that California would be too tantalizing to resist. According to a spokesperson for Romney, the governor supports hESC research but not SCNT. He contends that only a few Massachusetts companies are actually focused on stem cell research, and hence he's not concerned about scientists migrating west en masse.

**Figure 2:**
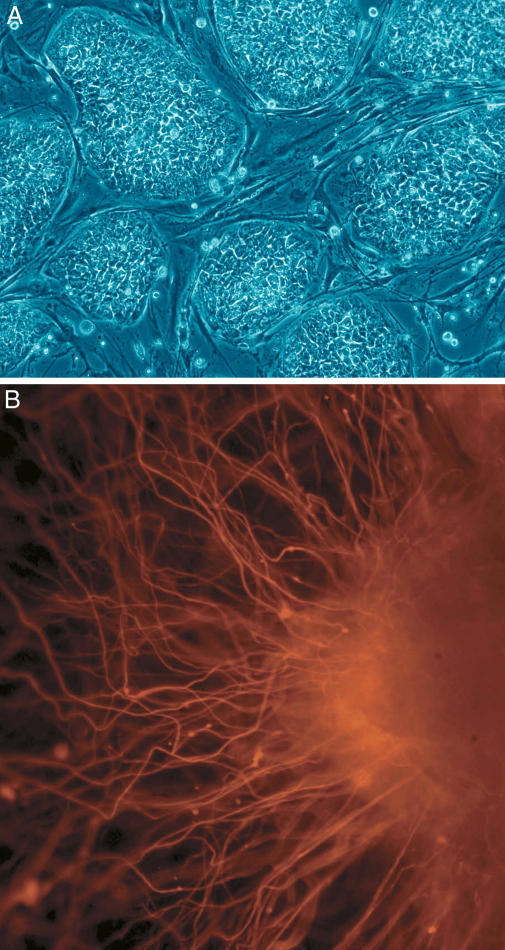
Embryonic Stem Cells (A) shows hESCs. (B) shows neurons derived from hESCs. (Images: Nissim Benvenisty)

In Maryland, a bill to fund hESC research was narrowly defeated in April of this year after months of political wrangling by a divided legislature. Like Governor Romney, Republican governor Robert Ehrlich supports, according to a spokesperson, embryonic stem cell research and President Bush's policy. But he has taken no official position on SCNT, nor on the recently defeated bill (which never actually got to his desk). Using money garnered from cigarette company restitution, the bill would have funneled US$25 million to embryonic stem cell research on discarded embryos, but would not have supported SCNT. Many in Maryland are keen to protect the state's life sciences investment. Already, though, California schools are making inquiries. “We certainly are having our share of people who are being recruited away at all levels,” says John Gearhart, a professor of biochemistry and molecular biology at Johns Hopkins University (Baltimore, Maryland, United States of America) and one of the first scientists to derive pluripotent human stem cells in 1998 (Figure 2). Gearhart is encouraged that the bill came within one vote of passing, and hopes that supporters will have better luck in the next legislative session. But he notes that time is running out; the Maryland legislature doesn't meet again until January of 2006, and any program that's approved would require a couple of years to ramp up.

Other states, actually acting prior to Prop 71 based on concerns about researchers doing any sort of cloning, therapeutic or otherwise, have enacted outright restrictions, mostly with regard to SCNT. Arkansas, North and South Dakota, Iowa, and Michigan all have laws specifically restricting aspects of hESC research. A Missouri state senate bill that would have criminalized SCNT recently stalled. In some states, including Texas, Illinois, and New York, lawmakers have proposed bills both for and against stem cell research.

## Best of a Bad Regulatory Situation

Despite the positive activity in many places, researchers like Gearhart and Weissman admit that it's only the best of a bad situation. A blanket federal funding policy would be much preferable. “I like to say that the glass is half full,” says Carl Gulbrandsen, director of the nonprofit WiCell Research Institute, which supports human embryonic stem cell research at the University of Wisconsin-Madison (Madison, Wisconsin, United States of America). Gulbrandsen distributes hESC lines derived by Wisconsin stem cell researcher Jamie Thomson, another pioneer of hESC research. It's encouraging, notes Gulbrandsen, that a large state like California, already suffering from severe deficit, has supported stem cell research. And Prop 71 has raised the value of the research in terms of licensing opportunities, with companies, both biotechs and pharmas, showing increased interest.

But Prop 71 also promises to increase the cost of doing research by making the attraction and retention of faculty more expensive as California and potentially other states offer millions in grants and salaries to stem cell researchers. “It's going to be very difficult,” says Gulbrandsen. “We're going to have to open the pocketbook and spend a lot of money to make sure the research goes forward.” In general, the state-by-state funding model “is a very poor public policy,” he adds. “If you end up with a patchwork of regulations, you're going to both decrease the movability of scientists and you're going to discourage collaborations.” Gulbrandsen suggests that intellectual property could be an issue as well, with states passing their own regulations, controlling their own intellectual property, and hence potentially creating turf wars among themselves and conflicts with federal policy. He says, “It's not a good road to walk down.”

Johns Hopkins president William Brody, though he supported funding efforts in Maryland, calls the growing influence of state legislators on the lab “scientific tourism.” “It's just going to create havoc about what you can do where, and I don't think in the end it's good science policy or good health care policy,” he says, noting that many states have actually restricted the research, in effect putting in place harsher regulations than those of the federal government. And regulations in other types of research could follow, Brody contends.

The quality of the research is at issue as well. Brody worries that smaller states won't be able to replicate rigorous grant peer review. Weissman is most concerned that the considerable monies available could lead to the wasting of funding on sub-par research. He advocates carrying the money forward until it can be well spent, perhaps on expensive clinical trials that are years away.

## The Proposition Heard around the World

Meanwhile, researchers overseas are taking notice of Prop 71, also with a mix of support and trepidation. “There's been a little bit of complacency in Europe because of the Bush decree,” says Austin Smith, director of the Institute for Stem Cell Research at the University of Edinburgh (Edinburgh, United Kingdom), and coordinator of EuroStemCell (Edinburgh, United Kingdom), an international consortium for stem cell research funded by the European Union. “European institutions have got to get their act together, because otherwise all our best people will go to California or other states setting up programs.” Alan Trounson, director of Monash Institute of Reproduction and Development in Clayton, Australia, has little doubt that California's stem cell funding riches will drain away talent from down under. “It will certainly be a very attractive place for young people and mid-career people to go,” he says, noting that Prop 71 should help give young stem cell investigators confidence that their field has a secure future.

Laws regulating hESC research in Europe and around the world vary considerably. Countries like the United Kingdom, Sweden, and Singapore are among the most liberal. In the UK, researchers are allowed to use embryonic stem cells from discarded embryos, and they're allowed to create embryos for the purposes of research. The approval process, however, can be laborious, according to Smith, though it has improved considerably in the last couple of years. Researchers must get approval from their local hospital ethical board, then they must apply for a license from the Human Fertilisation and Embryology Authority. HFEA inspects the quality of the science, the researcher's justification for using human embryos, whether scientists are complying with the law, and whether they're following correct patient consent procedures. Once a license is granted, HFEA conducts yearly inspections to insure that every embryo is accounted for.

In the beginning of April 2005, Sweden's government specifically approved of producing embryonic stem cell lines using SCNT. Using hESCs from leftover embryos had already been allowed. Protections in place are akin to those of the UK. “I'm very happy with the Swedish law,” says Outi Hovatta, a stem cell researcher at the Karolinska Institutet in Stockholm, Sweden. “There are clear regulations. We know what we can do, and we know what we can't do.”

After a year of public education outreach that included discussions with bioethicists, scientists, and religious leaders, Singapore, having already invested substantial federal funds into the life sciences, established specific hESC regulations.

**Figure 3:**
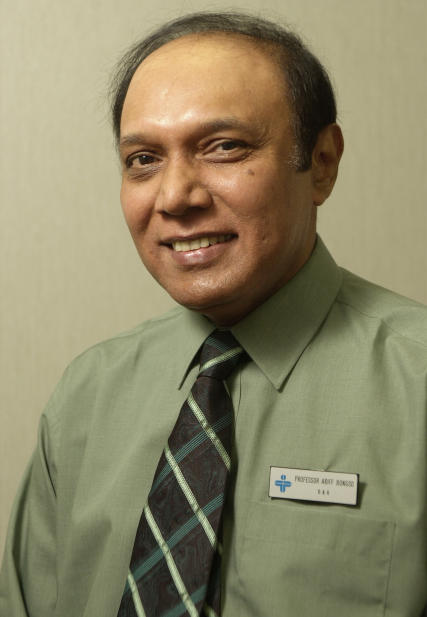
Ariff Bongso Ariff Bongso is a professor of obstetrics and gynecology and a pioneer of human embryonic stem cell research at the National University of Singapore.

The rules allow research on surplus in vitro fertilization (IVF) embryos using federal funding, and allow SCNT on a case by case basis. They also prohibit reproductive cloning; anyone caught attempting reproductive cloning receives a S$100,000 fine and ten years in jail. “I've never seen so much support from a government when it comes to stem cell biology,” Ariff Bongso, a professor of obstetrics and gynecology at the National University of Singapore (Figure 3). Bongso was the first person to isolate embryonic stem cells from human embryos in 1994. To derive hESCs, researchers must go to the hospital institutional review board, then to the government ministry of health, then to a government advisory bioethical council. Stem cell excitement has reached a fever pitch in Singapore, according to Bongso. “Everybody's talking about stem cells,” he says.

Countries like Israel and Australia have somewhat liberal laws, thought not quite as permissive. In Australia, laws used to be state by state, and cell lines could only be harvested from IVF embryos frozen prior to 2002. But a federal law now permits research on any frozen embryo, with the usual consent and ethical review. For now, SCNT has not been endorsed. In Israel, researchers can use embryonic stem cells from frozen embryos, but egg donation is not allowed, making SCNT all but impossible. Nissim Benvenisty, a stem cell researcher at the Hebrew University of Jerusalem (Jerusalem, Israel), says that many families are happy to donate discarded embryos that have been diagnosed with genetic diseases so that researchers can study diseased cell development. Countries like Switzerland, Spain, and France allow stem cell research with some restrictions.

Austria and Germany are much more restrictive. Brustle attributes his country's regulations to religious views, a legacy of eugenics, and negative attitudes towards new technologies. Brustle finds it difficult to conduct collaborations and he finds funding set-up somewhat nonsensical. For example: The European Commission, which will fund established stem cell lines, has a more liberal policy than Germany. Germany's European Commission stem cell research contribution ends up being funneled to countries with more liberal policies. Germany, then, indirectly pays for research in other countries that it actually deems illegal.

European collaborative efforts, though, continue to pick up steam. EuroStemCell, organized via the European Union, has 27 different member labs throughout Europe, with interests in adult and embryonic stem cells, clinical and basic research. According to Smith, the organization's charge is the exchange of materials and people. Smith would like to build up a cadre of European researchers trained in stem cell biology. “We have to collaborate in Europe to be competitive with the US,” he says. “It's not so clear that that's an imperative if you're sitting in California with all that money.”

## Abbreviations

hESC: human embryonic stem cell
HFEA: Human Fertilisation and Embryology Authority
IVF: in vitro fertilization
NIH: National Institutes of Health
SCNT: somatic cell nuclear transfer
